# Education Research: Neuroradiology Curriculum and Competencies Among Canadian Adult Neurology Residency Programs: A Cross-Sectional Study

**DOI:** 10.1212/NE9.0000000000200096

**Published:** 2023-11-06

**Authors:** Diana Benea, Rose Di Ioia, Julien Bejjani, Anne Xuan-Lan Nguyen, Isabelle Hardy, Isabelle Trop, Nicolas Jodoin

**Affiliations:** From the School of Medicine (D.B., R.D.I., A.X.-L.N.), Faculty of Medicine and Health Sciences, McGill University; Department of Radiology, Radiation Oncology and Nuclear Medicine (J.B., I.T.), Faculty of Medicine, Université de Montréal; Department of Ophthalmology (I.H.), Faculty of Medicine, Université de Montréal; Department of Diagnostic Radiology (I.T.), Centre Hospitalier Universitaire de Montréal; and Department of Neurosciences (N.J.), Faculty of Medicine, Université de Montréal, Montreal, Canada.

## Abstract

**Background and Objectives:**

While benefitting from neuroradiologists' reports, neurologists use their own image interpretation to guide clinical decisions, especially in acute care settings. This calls for robust neuroradiology training in neurology residency, informed by current educational gaps and practices. This study aims to (1) characterize the formal neuroradiology curriculum among Canadian neurology residency programs; (2) assess neurology residents' neuroimaging interpretation competencies; and (3) define neurology residents' and program directors' (PDs) attitudes toward the current curriculum and future directions.

**Methods:**

Anonymous surveys were sent to Canadian neurology residents and PDs, querying neuroradiology learning activities, imaging modalities covered, assessment modalities, perceived residents' competencies to interpret different modalities, and attitudes regarding neuroradiology training. Residents were asked to interpret 15 neuroimaging cases. Descriptive and inferential analyses were performed. Potential differences in residents' interpretation success rates by seniority, self-perceived proficiency, and perception of curriculum sufficiency were examined using 2-tailed Welch tests with a 95% CI and Holm-Bonferroni comparison adjustment. Statistics were computed using Excel.

**Results:**

Seventy-eight (32.6%) residents and 11 (68.8%) PDs participated. Ten of 11 PDs reported including a mandatory neuroradiology rotation, and 9/11 offered a formal neuroradiology curriculum covering head CT, head and neck CT angiography (CTA), spine MRI, and head MRI. Programs predominantly offered additional didactic lectures (9/11), teaching cases (8/11), and imaging websites (8/11). Most of the residents agreed with a minimum 1-month long rotation and desired regular didactics from neuroradiologists. Residents favored learning about head MRI (88.5%), head and neck CTA (76.9%), and spine MRI (69.2%). Senior residents' self-perceived competencies were highest for head CT, head MRI, and head and neck CTA, but lower than PDs' perception. Senior residents had greater interpretation scores than juniors (84.5% ± 13.2% vs 69.1% ± 19.9%; *p* < 0.0001). Most PDs (7/11, 63.6%) expressed satisfaction with current curricula vs 32.1% of residents. PDs identified time and educator shortages as main barriers to increased training.

**Discussion:**

Neuroradiology training varies among programs. Residents expressed strong interest in commonly taught modalities, for which they also expressed high self-perceived competencies. However, PDs expressed greater satisfaction than residents with the current training. Leveraging interactions with neuroradiologists and online case-based learning while emphasizing trainees' interests can enhance postgraduate neuroradiology training for this useful skill.

## Introduction

With advances in neuroimaging technology over the past few decades, neuroimaging has become an integral part of diagnosis, prognostication, and management in daily neurology practice. While greatly benefitting from trained neuroradiologists' reports,^1^ neurologists often also rely on their own interpretation^2,3^ to guide clinical decisions due to clinical knowledge that may influence studies' interpretation or necessity for rapid decision-making in acute settings. Therefore, neuroimaging interpretation is an essential skill for neurologists to optimize patient care.

Since the nationwide transition of Canadian neurology residency programs to a competence by design (CBD) model in July 2020, stages of training are marked by the achievement of specific competencies and comprise milestones and entrustable professional activities (EPAs), which guide neurology residency curricula. In the published *2020 Neurology Competencies* by the Royal College of Physicians and Surgeons of Canada (RCPSC),^4^ neuroimaging comprises its own competence and includes subpoints about anatomy, pathophysiology, indications, contraindications, and limitations of neuroimaging. Only the modalities of magnetic resonance studies and functional neuroimaging are specifically mentioned. In the *2020 Entrustable Professional Activities for Adult Neurology* by the RCPSC, “selecting, interpreting, and ordering appropriate neuroimaging” among other investigations is found as a feature of “EPA #1A: assessing and initiating management for patients with neurologic emergencies.”^5^ The ability to interpret relevant neuroimaging diagnostic studies is also assessed in the RCPSC end-of-training examination, but the proportion of imaging questions is not specified.^6^

Beyond the abovementioned references to neuroimaging in published RCPSC documentation guiding neurology curricula, specific learning objectives pertaining to neuroimaging modalities and pathologies to be mastered by neurology residents in CBD have not been well-described in Canada. A review of official neurology program descriptions^7^ further reveals that neuroradiology educational activities, including clinical radiology rotations, vary among Canadian neurology programs. A clearly defined formal neuroradiology curriculum for neurology, encompassing learning objectives with corresponding educational activities and assessments within the CBD framework has yet to be established. Moreover, Canadian neurology residents' competencies in interpreting neuroimaging studies at various stages of their training should be further explored. Finally, little is known about the current formal radiology training among Canadian neurology residency programs and residents' and program directors' (PD) attitudes toward this issue. Addressing this constitutes a steppingstone toward the implementation of a structured neuroradiology curriculum, bridging the current educational gap and promoting consistency in the learning outcomes achieved by neurology residents across Canada.

In this study, we aim to (1) characterize the formal neuroradiology curriculum among Canadian neurology residency programs; (2) assess neuroimaging interpretation competencies of Canadian neurology residents; and (3) define Canadian neurology residents' and PDs' attitudes toward the current radiology curriculum and future directions.

## Methods

A cross-sectional survey-based study was conducted among Canadian adult neurology residency programs. Two surveys, available in English and French, were created using Google Forms to collect data from a convenience sample of Canadian neurology residents and PDs.

The survey dedicated to neurology PDs was composed of 22 multiple-choice and short answer questions (eAppendix 1, links.lww.com/NE9/A48). It queried whether their program offered a formal neuroradiology curriculum, the curriculum's timing of delivery, and imaging modalities covered. The survey also collected PDs' affiliated institution, neuroradiology educational activities and assessment modalities, their impression of senior residents' competency to interpret various neuroimaging modalities and of the current curriculum, and barriers to increasing neuroradiology training. The year-of-training defining seniority was left to the PDs' discretion in the survey because it may vary between programs. Senior residents assume greater on-call responsibilities, including imaging interpretation skills. For analysis purposes, we used postgraduate year (PGY)3 as the transition year to senior resident to account for most programs.

The first section of the survey dedicated to neurology residents (eAppendix 2, links.lww.com/NE9/A48) included 21 multiple-choice and short answer questions and gathered data about residents' demographics (institution name, level of training), current neuroradiology training (educational resources available and used, exposure to relevant experts), self-assessed competency to interpret neuroimaging modalities, and impressions regarding their desired neuroradiology teaching. The second section of the survey comprised 15 multiple-choice neuroimaging diagnostic questions.

Survey questions were developed by authors D.B. and R.D.I., fourth-year medical students, based on review of the current literature^8-10^ and study objectives. To develop the imaging questions, D.B. and R.D.I. referred to images presented in the 2022 Neurology Residency In-Service Training Examination (RITE) Discussion and Reference Manual^11^ to create a list of 21 acute and chronic pathologies; RITE questions were not available to the authors. Corresponding clinical vignettes were written, and images were sourced with permission from Radiopaedia.org by D.B., R.D.I., and third-year radiology resident J.B. Modalities deemed most commonly used and referenced in the American Accreditation Council for Graduate Medical Education (ACGME) Neurology milestones^10^ and previous studies^8^ were chosen to be included in the exercise: head CT, head MRI, and spine MRI. Head CT angiography (CTA) was omitted because of interpretive challenges with static images. Multiple-choice lists were created using clinical judgment and the pathology-specific differential diagnosis list on Radiopaedia.org.

Experts I.T. and N.J., who are Radiology and Neurology Program Directors with academic appointments at the University of Montreal, respectively, used the concept of face validity to ascertain the clarity and appropriateness of the survey in an open-ended qualitative format. Question choices were modified after feedback. Fifteen image interpretation questions were retained. The survey was subsequently piloted with a third-year neurology resident and a third-year diagnostic radiology resident. After pilot testing, contextual biases associated with inclusion of clinical vignettes in interpretation questions were highlighted. Clinical vignettes were therefore omitted to assess residents' radiologic competencies without potential confounders of clinical knowledge. Moreover, ambiguous images and multiple choices deemed too challenging were replaced. Experts I.T. and N.J. reviewed the final versions of the surveys prior to survey release.

All 16 Canadian adult neurology program coordinators received open-access links to the surveys via published email addresses^7^ and were asked to forward them to adult neurology residents and to the PD. Respondents were able to review their answers prior to submission. Reminders were sent to program coordinators, program resident representatives, and PDs whose contact information was available online. Data were initially collected in November 2022 over 4 weeks, yielding a 19% resident response rate and 50% PD response rate. On initial peer-review of the study, this sample was not deemed representative. To increase representation from Canadian neurology programs, both resident and PD surveys were again released in April 2023 for 3 weeks. After data collection ended, the resident sample surpassed the minimum size (n = 69) determined using the finite population correction formula with a desired 95% confidence level and 10% margin of error. The Canadian adult neurology resident population size (N = 239) was calculated using published yearly quota.^12^

All submitted questionnaires were fully completed. All submissions occurred at least 15 minutes after survey distribution, the estimated time required for completion. Data were automatically compiled in a password-protected spreadsheet, which will be stored for the next 7 years. Data were analyzed by D.B. and R.D.I. A descriptive analysis was performed using mean values and SDs for continuous variables and frequencies for categorical variables. Inferential analyses comparing residents' imaging interpretation success rates by resident seniority, senior residents' perception of the current curriculum's sufficiency, and head CT and MRI self-perceived competency were performed. Given assumption of unequal variances between groups, 2-tailed Welch *t* tests were computed with α = 0.05. Four comparisons were made; *p* values were adjusted using the Holm-Bonferroni method. Statistics were computed using Microsoft Excel (Microsoft Corp., Redmond, WA).

### Standard Protocol Approvals, Registrations, and Patient Consents

Ethical approval for the study, led by authors A.X.-L.N. and I.H., was granted by the University of Montreal's Institutional Review Board (*Comité d’éthique de la recherche clinique*; Project 2022-1654). Each survey included a consent form with the study's description, benefits, risks, confidentiality statement, study withdrawal clarifications, and contact information. Implicit consent was obtained with a checkbox question allowing participants to proceed to the main survey. Anonymous data collection ensured confidentiality.

### Data Availability

Anonymized data not provided in the article may be shared on request of qualified investigators for replication purposes.

## Results

All 16 Canadian adult neurology programs participated in the study. Data were obtained from 78 (32.6%) residents and 11 (68.8%) PDs in adult neurology.

### Program Director Survey

#### Current Neuroradiology Curriculum

Among 11 participating PDs (eTable 1, links.lww.com/NE9/A50), 9/11 (81.8%) reported offering a formal neuroradiology curriculum, delivered either longitudinally (2/9, 22.2% of programs with a formal curriculum), in junior years (PGY1/PGY2) (3/9, 33.3%), in senior years (PGY3/PGY4) (3/9, 33.3%), or in both PGY2 and PGY4 (1/9, 11.1%). The curricula covered head CT, head and neck CTA, head MRI, and spine MRI. Among those, 5/9 (55.6%) programs also included education on cerebral perfusion scans, 2/9 (22.2%) on conventional catheter angiography, and 1/9 (11.1%) on nuclear imaging. However, no program included transcranial or carotid Doppler ultrasounds.

In 90.9% (10/11) of programs, a mandatory neuroradiology rotation was offered, lasting 4 weeks (5/10, 50%), 4–8 weeks (4/10, 40%), or more than 8 weeks (1/10, 10%). Other neuroradiology educational resources included didactic lectures (9/11, 81.8%), teaching cases (8/11, 72.7%), imaging websites (8/11, 72.7%), neuroradiology conferences (4/11, 36.4%), online lectures (3/11, 27.3%), and flash cards (1/11, 11.1%).

Formal assessment of residents' neuroradiologic competencies was conducted in 9/11 (81.8%) participating programs. Of those, 7/9 (77.8%) programs used direct observation in the clinical setting (e.g., EPA or on-call code stroke clinical evaluation) as a main assessment modality. Five of 9 (55.6%) programs used simulation-based sessions (e.g., objective structured clinical examinations), while 4/9 (44.4%) programs used written examinations and 3/9 (33.3%) used oral presentations. Five of 9 programs (55.6%) with formal assessments endorsed using a combination of modalities.

#### Image Interpretation: Perceived Competency

**Figure 1:**
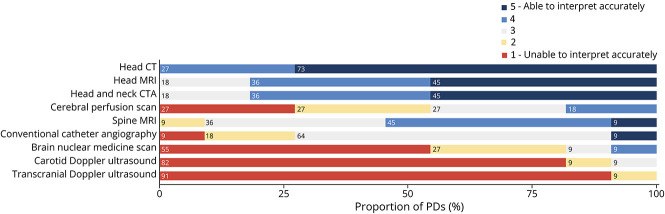
PDs' Perceived Senior Residents' Competencies to Interpret Neuroimaging Modalities CTA = CT angiography; PD = program director.

Using a 5-point Likert scale, PDs ranked their perception of senior residents' interpretation skills for different neuroimaging modalities (1 = unable, 5 = able to interpret accurately) (Figure 1). All PDs believed senior residents could accurately interpret (ranked at 4 or 5) head CTs. Most of the PDs felt seniors could accurately interpret head and neck CTAs (9/11, 81.8%), head MRIs (9/11, 81.8%), and spine MRIs (6/11, 54.5%). Perceived cerebral perfusion scan interpretation skills varied greatly, with 27% (3/11) of PDs ranking at 1, 2, and 3 each and 18.2% of PDs (2/11) ranking at 4. Most of the PDs (7/11, 63.6%) perceived conventional catheter angiography interpretation skills at 3. However, 81.8% (9/11) of PDs ranked senior residents' brain nuclear medicine scan (e.g., PET) interpretation skills at 2 or 1. Finally, seniors were thought to have poor interpretation skills in carotid and transcranial Doppler ultrasounds, with most PDs rankings at 1 for both (9/11, 81.8% and 10/11, 90.9%, respectively).

#### Attitudes Regarding the Neuroradiology Curriculum

**Figure 2:**
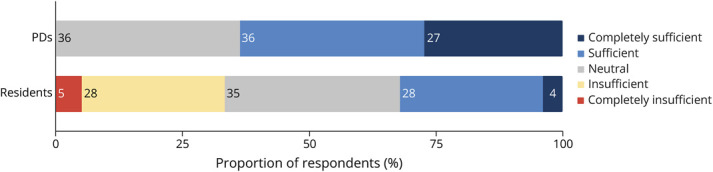
PDs' and Residents' Perception of the Current Neuroradiology Curriculum PD = program director.

Most of the PDs considered residents' neuroradiology training to be sufficient (Figure 2). No programs deemed it insufficient nor excessive.

PDs were asked to rank potential barriers to increasing neuroradiology training in their program in order of importance. According to weighed ranks, lack of time, followed by lack of educators and lack of high-quality educational material were the most important barriers (eFigure 1, links.lww.com/NE9/A49). Lack of trainee interest was ranked as least important overall. One PD suggested optimizing training during neuroradiology rotations because it may not be fully catered toward neurology residents' learning objectives and expressed interest in a national neuroradiology curriculum. Another specified lack of “willing” educators in the radiology department as a barrier.

### Resident Survey

#### Radiology Didactics

Residents from 15/16 programs participated in the study (eTable 1, links.lww.com/NE9/A50). Most of the resident respondents were in PGY2 (28.2%), followed by PGY1 and PGY4 (20.5% each), PGY3 (17.9%), and PGY5 (12.8%). During neurology rotations, 59% of residents disclosed spending less than 15 minutes weekly reviewing images with a neuroradiologist and reported interactions were limited to a cumulative weekly maximum of 2 hours. Meanwhile, most residents reported spending at least 1 cumulative hour reviewing scans with neurologists weekly. Some (17.9%) even reported spending 4 hours or more doing so (Table 1).

**Table 1:**
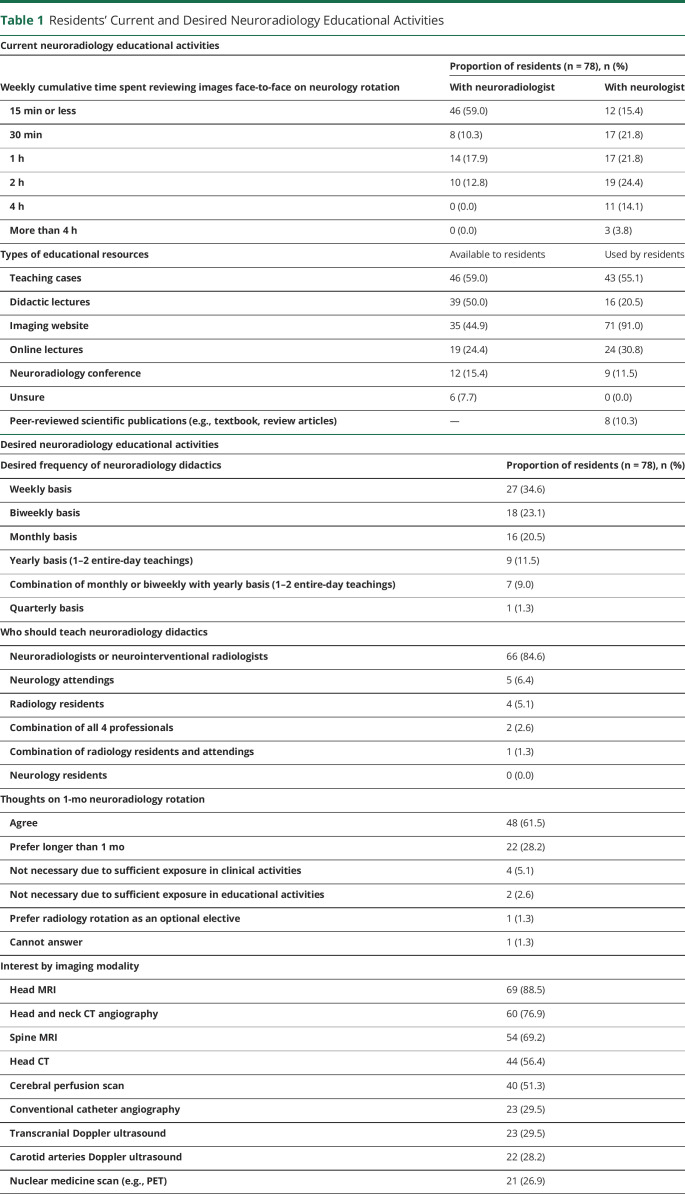
Residents' Current and Desired Neuroradiology Educational Activities

Current neuroradiology educational activities		
	Proportion of residents (n = 78), n (%)	
Weekly cumulative time spent reviewing images face-to-face on neurology rotation	With neuroradiologist	With neurologist
15 min or less	46 (59.0)	12 (15.4)
30 min	8 (10.3)	17 (21.8)
1 h	14 (17.9)	17 (21.8)
2 h	10 (12.8)	19 (24.4)
4 h	0 (0.0)	11 (14.1)
More than 4 h	0 (0.0)	3 (3.8)
Types of educational resources	Available to residents	Used by residents
Teaching cases	46 (59.0)	43 (55.1)
Didactic lectures	39 (50.0)	16 (20.5)
Imaging website	35 (44.9)	71 (91.0)
Online lectures	19 (24.4)	24 (30.8)
Neuroradiology conference	12 (15.4)	9 (11.5)
Unsure	6 (7.7)	0 (0.0)
Peer-reviewed scientific publications (e.g., textbook, review articles)	—	8 (10.3)

Desired neuroradiology educational activities	
Desired frequency of neuroradiology didactics	Proportion of residents (n = 78), n (%)
Weekly basis	27 (34.6)
Biweekly basis	18 (23.1)
Monthly basis	16 (20.5)
Yearly basis (1–2 entire-day teachings)	9 (11.5)
Combination of monthly or biweekly with yearly basis (1–2 entire-day teachings)	7 (9.0)
Quarterly basis	1 (1.3)
Who should teach neuroradiology didactics	
Neuroradiologists or neurointerventional radiologists	66 (84.6)
Neurology attendings	5 (6.4)
Radiology residents	4 (5.1)
Combination of all 4 professionals	2 (2.6)
Combination of radiology residents and attendings	1 (1.3)
Neurology residents	0 (0.0)
Thoughts on 1-mo neuroradiology rotation	
Agree	48 (61.5)
Prefer longer than 1 mo	22 (28.2)
Not necessary due to sufficient exposure in clinical activities	4 (5.1)
Not necessary due to sufficient exposure in educational activities	2 (2.6)
Prefer radiology rotation as an optional elective	1 (1.3)
Cannot answer	1 (1.3)
Interest by imaging modality	
Head MRI	69 (88.5)
Head and neck CT angiography	60 (76.9)
Spine MRI	54 (69.2)
Head CT	44 (56.4)
Cerebral perfusion scan	40 (51.3)
Conventional catheter angiography	23 (29.5)
Transcranial Doppler ultrasound	23 (29.5)
Carotid arteries Doppler ultrasound	22 (28.2)
Nuclear medicine scan (e.g., PET)	21 (26.9)

In neurology training programs, teaching cases (59%), didactic lectures (50%), and imaging websites (44.9%) supplement neuroradiology learning. Although most of the residents use teaching cases (55.1%), a greater proportion (91%) opt for imaging websites (Table 1). Most of the residents preferred neuroradiology didactics to be held on a weekly (34.6%), biweekly (23.1%), or monthly basis (20.5%) and led by neuroradiologists or neurointerventional radiologists (84.6%) (Table 1).

#### Attitudes Regarding the Neuroradiology Curriculum

No resident thought the neuroradiology teaching received in their program was excessive. Perceptions of curriculum sufficiency were divided because 32.1% of residents found the neuroradiology training to be sufficient and 33.3% insufficient (Figure 2). The proportion of senior residents was higher among those who perceived the curriculum as sufficient than those who perceived it as insufficient (68% vs 38.5%). Most of the residents (61.5%) agreed that a 1-month neuroradiology rotation is important to complement their training, and notably, 28.2% of residents preferred a longer rotation (Table 1). Residents were asked to select the neuroimaging modalities they were most interested in learning about from a list and most often chose head MRI, followed by head and neck CTA, spine MRI, head CT, and cerebral perfusion scans (Table 1).

#### Image Interpretation: Self-Perceived and Measured Competency

**Figure 3:**
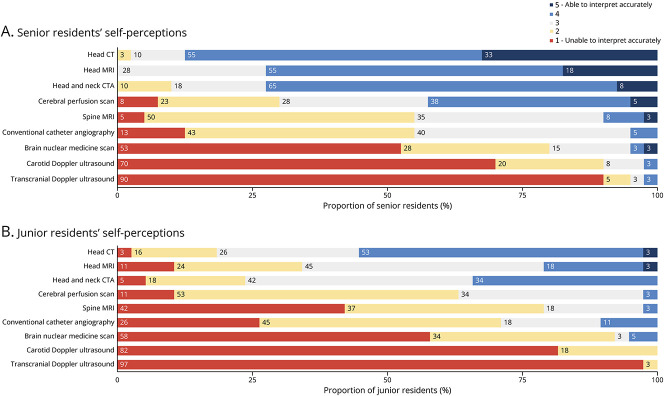
Senior and Junior Residents' Self-Perceived Competencies to Interpret Neuroimaging Modalities CTA = CT angiography.

Similar to PDs, residents were asked to rank their self-perceived competency to interpret various neuroimaging modalities (Figure 3). When looking at senior residents' responses, head CT interpretation skills were ranked highest among modalities, with 24 (87.5%) residents selecting a score of 4 or 5. Most of the senior residents' self-perceived competencies to interpret head MRI and head and neck CTA were ranked high at 4 or 5 (72.5% each). However, they had a lower perception of their spine MRI competency level, with 55% ranking at 1 or 2. For cerebral perfusion scans, 42.5% of residents ranked their skills at 4 or 5 and 30% at 1 or 2. Lastly, a significant majority believed being unable to accurately interpret nuclear imaging and carotid and transcranial Doppler ultrasounds. Most of the junior residents believed they were able to accurately interpret head CT (55.3%), while a smaller proportion did for head MRI (21.1%) and head and neck CTA (34.2%). Most of the junior residents thought they were unable to accurately interpret spine MRIs (78.9%) and cerebral perfusion scans (63.2%).

When given a series of 15 neuroimaging questions with multiple-choice answers (Table 2), senior residents had significantly greater success rates than junior residents (mean ± SD; 84.5% ± 13.2% vs 69.1% ± 19.9%, *p* < 0.0001). Given the greater proportion of senior residents among those who thought the curriculum to be sufficient, differences in image interpretation rates between residents who believed the curriculum to be sufficient vs insufficient were tested for significance among senior residents only. Senior residents who believed the curriculum to be sufficient had higher interpretation success rates than seniors who did not (89.8% ± 13.8% vs 77.3% ± 22.8%, *p* = 0.0145). Residents with higher self-perceived competency for head CT and MRI had statistically significant higher interpretation success rates for the respective modality (CT: 90.4% ± 15.7% vs 62.5% ± 19.8%, *p* = 0.0111; MRI: 79.3% ± 17.7% vs 65.6% ± 14.2%, *p* = 0.0253) (Table 3).

**Table 2:**
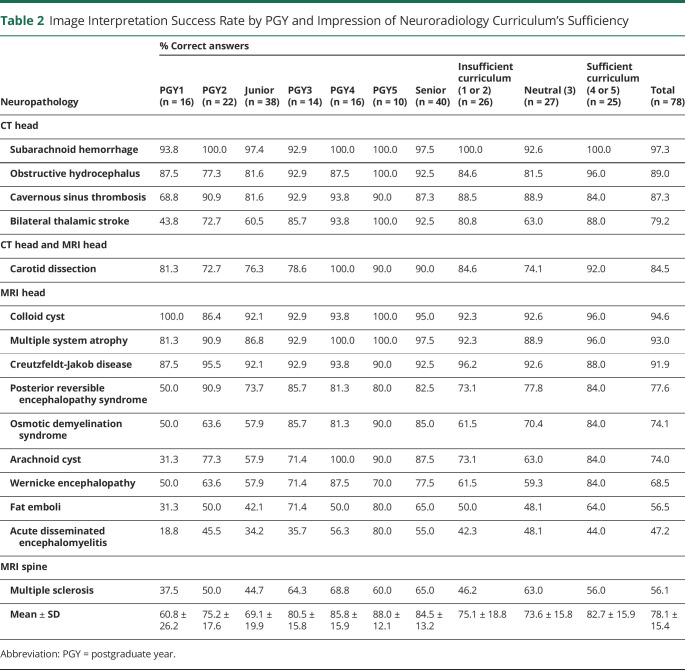
Image Interpretation Success Rate by PGY and Impression of Neuroradiology Curriculum's Sufficiency

Neuropathology	% Correct answers										
	PGY1 (n = 16)	PGY2 (n = 22)	Junior (n = 38)	PGY3 (n = 14)	PGY4 (n = 16)	PGY5 (n = 10)	Senior (n = 40)	Insufficient curriculum (1 or 2) (n = 26)	Neutral (3) (n = 27)	Sufficient curriculum (4 or 5) (n = 25)	Total (n = 78)
CT head											
Subarachnoid hemorrhage	93.8	100.0	97.4	92.9	100.0	100.0	97.5	100.0	92.6	100.0	97.3
Obstructive hydrocephalus	87.5	77.3	81.6	92.9	87.5	100.0	92.5	84.6	81.5	96.0	89.0
Cavernous sinus thrombosis	68.8	90.9	81.6	92.9	93.8	90.0	87.3	88.5	88.9	84.0	87.3
Bilateral thalamic stroke	43.8	72.7	60.5	85.7	93.8	100.0	92.5	80.8	63.0	88.0	79.2
CT head and MRI head											
Carotid dissection	81.3	72.7	76.3	78.6	100.0	90.0	90.0	84.6	74.1	92.0	84.5
MRI head											
Colloid cyst	100.0	86.4	92.1	92.9	93.8	100.0	95.0	92.3	92.6	96.0	94.6
Multiple system atrophy	81.3	90.9	86.8	92.9	100.0	100.0	97.5	92.3	88.9	96.0	93.0
Creutzfeldt-Jakob disease	87.5	95.5	92.1	92.9	93.8	90.0	92.5	96.2	92.6	88.0	91.9
Posterior reversible encephalopathy syndrome	50.0	90.9	73.7	85.7	81.3	80.0	82.5	73.1	77.8	84.0	77.6
Osmotic demyelination syndrome	50.0	63.6	57.9	85.7	81.3	90.0	85.0	61.5	70.4	84.0	74.1
Arachnoid cyst	31.3	77.3	57.9	71.4	100.0	90.0	87.5	73.1	63.0	84.0	74.0
Wernicke encephalopathy	50.0	63.6	57.9	71.4	87.5	70.0	77.5	61.5	59.3	84.0	68.5
Fat emboli	31.3	50.0	42.1	71.4	50.0	80.0	65.0	50.0	48.1	64.0	56.5
Acute disseminated encephalomyelitis	18.8	45.5	34.2	35.7	56.3	80.0	55.0	42.3	48.1	44.0	47.2
MRI spine											
Multiple sclerosis	37.5	50.0	44.7	64.3	68.8	60.0	65.0	46.2	63.0	56.0	56.1
Mean ± SD	60.8 ± 26.2	75.2 ± 17.6	69.1 ± 19.9	80.5 ± 15.8	85.8 ± 15.9	88.0 ± 12.1	84.5 ± 13.2	75.1 ± 18.8	73.6 ± 15.8	82.7 ± 15.9	78.1 ± 15.4

Abbreviation: PGY = postgraduate year.

**Table 3:**
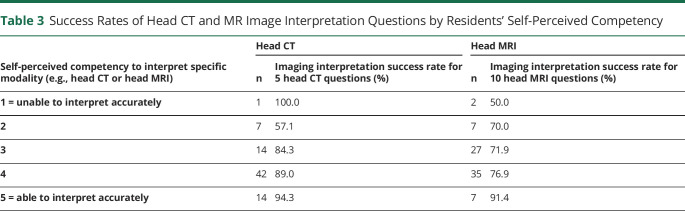
Success Rates of Head CT and MR Image Interpretation Questions by Residents' Self-Perceived Competency

Self-perceived competency to interpret specific modality (e.g., head CT or head MRI)	Head CT		Head MRI	
	n	Imaging interpretation success rate for 5 head CT questions (%)	n	Imaging interpretation success rate for 10 head MRI questions (%)
1 = unable to interpret accurately	1	100.0	2	50.0
2	7	57.1	7	70.0
3	14	84.3	27	71.9
4	42	89.0	35	76.9
5 = able to interpret accurately	14	94.3	7	91.4

## Discussion

We examined the educational activities and assessment modalities of neuroradiology curricula in Canadian neurology residency programs and described neurology residents' and PDs' perceived and expressed needs regarding postgraduate neuroradiology training. In addition, we attempted to assess residents' neuroimaging interpretation skills. One prior study surveying neurology residency PDs in the United States^8^ revealed that one-third of programs lacked a formal neuroimaging curriculum and half did not require a neuroradiology rotation. In our study, most participating Canadian neurology residency programs include a formal neuroradiology curriculum and all but 1 require a mandatory radiology rotation. However, heterogeneity regarding timing of delivery, imaging modalities, educational activities, and assessment leave the definition of “formal curriculum” in neuroradiology training without clear consensus. Although no PDs perceived the current neuroradiology training as insufficient, one-third of residents did. By integrating resident preferences and PDs' perceptions, our study yields specific recommendations for each component that may compose a formal neuroradiology curriculum (Table 4).

**Table 4:**
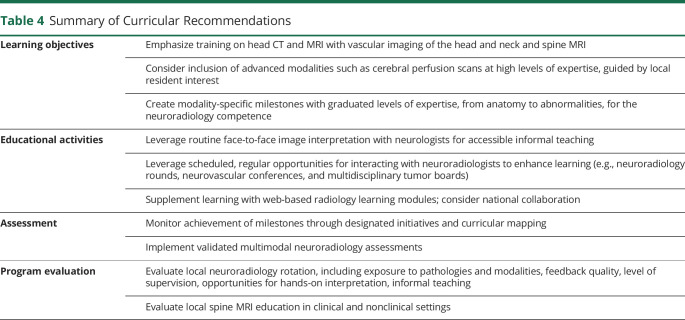
Summary of Curricular Recommendations

Learning objectives	Emphasize training on head CT and MRI with vascular imaging of the head and neck and spine MRI
	Consider inclusion of advanced modalities such as cerebral perfusion scans at high levels of expertise, guided by local resident interest
	Create modality-specific milestones with graduated levels of expertise, from anatomy to abnormalities, for the neuroradiology competence
Educational activities	Leverage routine face-to-face image interpretation with neurologists for accessible informal teaching
	Leverage scheduled, regular opportunities for interacting with neuroradiologists to enhance learning (e.g., neuroradiology rounds, neurovascular conferences, and multidisciplinary tumor boards)
	Supplement learning with web-based radiology learning modules; consider national collaboration
Assessment	Monitor achievement of milestones through designated initiatives and curricular mapping
	Implement validated multimodal neuroradiology assessments
Program evaluation	Evaluate local neuroradiology rotation, including exposure to pathologies and modalities, feedback quality, level of supervision, opportunities for hands-on interpretation, informal teaching
	Evaluate local spine MRI education in clinical and nonclinical settings

Given the variety of imaging modalities used in neurologic care, the choice of modalities included in neurology residency training is of interest. Participating programs uniformly covered commonly used modalities such as head CT, head and neck CTA, head MRI, and spine MRI in their curriculum, aligning with residents' interests. Conventional catheter angiographies, transcranial and carotid Doppler ultrasounds, and nuclear imaging were less prevalent in curricula, and PDs and residents reported low perceived competencies for them. Ultrasound-based images are considered operator dependent and reviewer dependent, which may present a barrier to Doppler ultrasound interpretation and education among neurology residents. However, neurosonology training is included in the ACGME accreditation requirements for neurology programs,^13^ while no modalities are mentioned in the RCPSC's requirements.^14^

The RCPSC competence tied to neuroimaging for Canadian neurology curricula does not detail modality-specific tasks^4^; neuroimaging-specific learning objectives within the CBD framework are needed. Notably, the ACGME 2021 Interpretation of Neuroimaging milestone lists specific tasks related to interpreting neuroimaging with graduated difficulty from novice to expert.^10^ Head CT and MRI with vascular imaging and spine CT and MRI are included, while “interpreting advanced imaging” is considered “expert” level. Our results support prioritizing the inclusion of head CT and MRI, including vascular imaging, and spine MRI in the curriculum. We suggest creating specific milestones focused on these prioritized modalities within the CBD framework, mirroring the ACGME's milestones with graduated levels from recognizing anatomy to increasingly challenging abnormalities. These would help guide corresponding EPAs and appropriate clinical and nonclinical learning opportunities for neuroimaging training. Forming a national working group to define these objectives may be considered.

Cerebral perfusion scan was a dividing modality; half of programs included training for it and half of residents expressed interest in it. It is the only modality for which residents rated their competency higher than PDs, although perceptions varied for both groups. This potentially reflects the modality's emerging significance due to its crucial use in contemporary acute stroke management, resulting in newer implementation among certain programs and fewer assessment data. Perfusion imaging processing varied between individuals with differing radiologic training, and neurology attendings had greater variability than radiologists or radiologists-in-training.^15^ Indeed, cerebral perfusion imaging interpretation is complex, requiring integration of software-generated quantitative metrics alongside head CT interpretation.^16-19^ This may influence PDs to be more conservative than residents in rating perceived competencies for this modality. Perfusion scans' increasing applicability should prompt educators to consider its inclusion in a future-forward neuroradiology curriculum.

All but 1 program mandated a neuroradiology rotation, in keeping with residents' preferences. However, PDs emphasized potential misalignment between training provided in radiology rotations and neurology residents' objectives. A limitation of our study is not querying residents' impressions of their radiology rotations. We recommend that programs evaluate neuroradiology rotations locally, including exposure to pathologies and modalities, feedback quality, supervision, informal teaching, and hands-on interpretation opportunities.

Reviewing images face-to-face with neuroradiologists or neurologists is a valuable component of neuroradiology training. In fact, neurointerventional rotations have been proposed to enhance neurology residents' interpretation of advanced imaging for prompt clinical decision-making in stroke management.^20^ However, our results reveal minimal weekly review time with neuroradiologists for most residents, mirroring results observed in previous studies.^8^ Conversely, most residents spend at least 1 hour face-to-face with neuroimaging-proficient neurologists weekly. Leveraging frequent image interpretation sessions led by neurologists represents a practical intervention to increase teaching. Since the implementation of the Picture Archiving and Communication System, face-to-face interactions between radiologists and consultants decreased,^3^ and this has been exacerbated by remote reporting during the coronavirus disease 2019 pandemic.^21^ Although this may pose a challenge for radiology teaching to consultants, collaboration between neurology and radiology departments may also occur via teleconference,^21^ which was not accounted for in our survey. Moreover, most residents wish to receive didactic neuroradiology teaching by neuroradiologists on a regular basis. However, only a fifth of residents report using didactic lectures to learn about neuroradiology despite lectures being offered by half of programs. This discrepancy may indicate that current didactic teaching offered does not meet residents' needs. Indeed, a lack of educators was noted by PDs as the second most important barrier to increasing neuroradiology training. As neurologists and radiologists work closely together clinically, this calls for extension of interprofessional collaboration in educational settings and recruitment of available neuroradiology educators. However, radiologists are facing greater image interpretation workloads due to increased image utilization rates,^22-24^ which represent a notable barrier to participation in educational responsibilities. Programs could nonetheless leverage existing opportunities such as neuroradiology rounds, neurovascular conferences, and multidisciplinary tumor boards to increase interactions with neuroradiology faculty and enhance resident learning, a recommendation also supported by previous studies.^8^

In addition to neuroradiology rotations and rounds, programs mostly made available teaching cases, didactic lectures, and imaging websites to residents. Despite residents being aware of educational materials offered by their program, most of them resorted to imaging websites, followed by teaching cases, with only a minority using online or didactic lectures to complement their learning. PDs cited access to high-quality educational material as the third most important barrier to increasing neuroradiology training. Computerized radiology learning modules can be effective at supplementing traditional radiologic teaching methods.^25-27^ Similarly, a previous study^8^ found web-based radiology resources could palliate perceived areas of weakness in neuroradiology curricula. For instance, the Association of University Radiologists established a cooperative Radiology Resident Core Curriculum Lecture Series; this standardized curriculum showcases virtual radiology learning programs' potential to enhance resident education across programs.^28^ Creating and sharing such materials nationally would address residents' online learning preferences, time restraints within curricula, and lack of available educators and educational materials.

Our study revealed differences between senior residents' and PDs' perception of residents' competencies. For common modalities such as head CT, head and neck CTA, head MRI, and spine MRI, PDs rated senior residents' neuroimaging interpretation competencies higher than senior residents did. Although not formally assessed, PDs' answers may have been positively skewed toward modalities included in their curriculum. This discrepancy was most pronounced for spine MRI, possibly reflecting lower exposure to spine MRI in neuroradiology educational activities and rotations than expected by PDs. We recommend that programs evaluate the frequency and quality of spine MRI teaching in their curriculum, including teaching cases, lectures, rounds, and rotations. Residents may also underestimate their proficiency; however, the significant association between high individual self-perceived proficiency and interpretation success rate for head CT and MRI suggests accurate resident self-evaluation. Variable concordance between resident and faculty assessment was found in the literature. Minor discrepancies were reported in general surgery assessment,^29^ while significant positive discrepancies were found for ophthalmology residents^30^ and emergency medicine residents^31^ self-assessments. By contrast, our study found negative discrepancies in resident self-assessment, except for perfusion imaging. Potential factors influencing discrepancies in resident self-assessment have been identified. For instance, previous studies reported that female trainees rated themselves lower than their male counterparts and were more likely to underestimate their skills.^29,32^ However, sex-based information was not collected in our study. Faculty may also assess graduating residents more generously because they are expected to have achieved high competency, while residents conservatively self-assess given “trepidation” as they finish training.^29^ Concordance of residents' self-assessment and underlying factors remain to be elucidated in future studies.

The discordance between PDs' and residents' perceived competencies may also reflect a gap in assessment strategies of residents' neuroradiologic competencies. Participating programs most assessed residents through direct observation in the clinical setting, followed by simulation-based activities and written examinations, but alignment with neurology residents' radiologic learning objectives is unclear. Validated, multimodal neuroradiology assessments, especially among programs without a formal assessment strategy or using a single modality, are needed. Monitoring achievement of competencies through designated initiatives such as curricular mapping of neuroradiology training can improve knowledge of residents' competencies and areas where support is needed.

This study also attempted to measure residents' image interpretation skills. However, our image interpretation questionnaire is an imperfect proxy for neuroradiology knowledge. Appropriate selection of neuroimaging modalities and their contraindications or indications was not tested. Although the survey was piloted, lacking validity evidence limits its results' generalizability. Questions do not precisely replicate real-life diagnostic challenges due to absent clinical information, multiple-choice answers, preselected image slides, pathognomonic imaging findings, and unambiguous images. Moreover, querying the perceived competencies by modality is not comprehensive of all possible pathologies and their varying diagnostic complexity. However, comprehensively assessing cases of widely varying complexity for each modality is impractical. We also believe gathering data by modality to be more informative for future curricula. It remains that although imperfect, our practical assessment contributes to addressing the knowledge gap about Canadian neurology residents' current neuroradiology skills. More comprehensive and validated assessment data covering all facets of residents' neuroradiologic competencies in clinical settings is needed.

While considering the limitations of the diagnostic exercise, our results indicate that senior residents performed significantly better at interpreting images than junior residents. Let it be reassuring for Canadian neurology educators that residents' neuroradiology interpretation skills appeared to improve with increased training. Senior residents who perceived neuroradiology training to be sufficient also had higher interpretation scores. Potential explanations include increased interest in neuroradiology among residents perceiving the curriculum as sufficient, leading to greater involvement in educational activities, or simply better neuroradiology training received. One can hypothesize that delivering adequate neuroradiology training leads to improvement in image interpretation skills, reinforcing the importance of adequate neuroradiology curricula.

The primary limitation of our study lies in the sample size of participating residents and the uneven representation of the 16 Canadian adult neurology programs, potentially skewing data toward overrepresented programs. Nonetheless, our study represents PDs from 11 and residents from all but 1 Canadian adult neurology programs. Nonresponse bias may also influence study results due to voluntary participation. Providing incentives or encouraging survey completion as part of curriculum-mandated educational activities may reduce such nonresponse bias in the future. Thorough survey pilot testing could have been conducted on a larger sample of neurology residents in various years of training. Another limitation of our study is not including imaging modalities such as peripheral nerve ultrasound and muscle MRI. In addition, “formal curriculum” was not clearly defined; thus, its interpretation may vary among PDs, regarding the timing of delivery. However, data we obtained about specific components of neuroradiology training help characterize individual programs' curricula. Querying neuroradiology-specific learning objectives for each program would have been a valuable addition to the survey and may be collected in future studies.

Most participating Canadian adult neurology residency programs reported having a formal neuroradiology curriculum and included a mandatory neuroradiology rotation. However, they remained variable in the timing of curriculum delivery, assessment, and resources offered. Modalities of greatest interest were covered and had the highest perceived competency rankings. PDs perceived spine MRI competencies markedly higher than senior residents did, prompting further evaluation of spine MRI training and accuracy of assessment strategies. Reassuringly, senior residents' interpretation success rates were significantly greater than junior residents' interpretation success rates. PDs expressed greater satisfaction with the neuroradiology curriculum than residents. Lack of time and educators were believed to be the main barriers to improving neuroradiology training. Residents valued formal neuroradiology curricula including mandatory neuroradiology rotations, regular teaching by neuroradiologists, and supplemental e-learning to enhance their training. Leveraging collaboration with neuroradiologists and neurologists can enhance postgraduate neuroradiologic education.

Residents' and PDs' expressed and perceived needs can inform the development of a neuroradiology curriculum for Canadian neurology residency programs in CBD. Further research to characterize teaching of this essential competency, including neuroradiology rotations' curricular alignment and residents' real clinical neuroradiology skills, is needed.

## Appendix Authors

**Table:**

Name	Location	Contribution
Diana Benea, MD	School of Medicine, Faculty of Medicine and Health Sciences, McGill University, Montreal, Canada	Drafting/revision of the article for content, including medical writing for content; major role in the acquisition of data; study concept or design; and analysis or interpretation of data
Rose Di Ioia, MD	School of Medicine, Faculty of Medicine and Health Sciences, McGill University, Montreal, Canada	Drafting/revision of the article for content, including medical writing for content; major role in he acquisition of data; study concept or design; and analysis or interpretation of data
Julien Bejjani, MD	Department of Radiology, Radiation Oncology and Nuclear Medicine, Faculty of Medicine, Université de Montréal, Montreal, Canada	Drafting/revision of the article for content, including medical writing for content; study concept or design
Anne Xuan-Lan Nguyen, MD	School of Medicine, Faculty of Medicine and Health Sciences, McGill University, Montreal, Canada	Drafting/revision of the article for content, including medical writing for content; study concept or design; ethics approval
Isabelle Hardy, MD, FRCSC	Department of Ophthalmology, Faculty of Medicine, Université de Montréal, Montreal, Canada	Drafting/revision of the article for content, including medical writing for content; study concept or design; ethics approval
Isabelle Trop, MD, FRCPC, MPH	Department of Radiology, Radiation Oncology and Nuclear Medicine, Faculty of Medicine, Université de Montréal, Montreal, Canada; Department of Diagnostic Radiology, Centre Hospitalier Universitaire de Montréal, Montreal, Canada	Drafting/revision of the article for content, including medical writing for content; study concept or design
Nicolas Jodoin, MD	Department of Neurosciences, Faculty of Medicine, Université de Montréal, Montreal, Canada	Drafting/revision of the article for content, including medical writing for content; study concept or design

## Glossary

ACGME: American Accreditation Council for Graduate Medical Education
CBD: competence by design
CTA: CT angiography
EPA: entrustable professional activity
PD: program director
PGY: postgraduate year
RCPSC: Royal College of Physicians and Surgeons of Canada
RITE: Residency In-Service Training Examination
